# Comparative gene expression profiling of mouse ovaries upon stimulation with natural equine chorionic gonadotropin (N-eCG) and tethered recombinant-eCG (R-eCG)

**DOI:** 10.1186/s12896-020-00653-8

**Published:** 2020-11-11

**Authors:** Kwan-Sik Min, Jong-Ju Park, So-Yun Lee, Munkhzaya Byambaragchaa, Myung-Hwa Kang

**Affiliations:** 1 Animal Biotechnology, Graduate School of Future Convergence Technology, Hankyong National University, Ansung, 17579 South Korea; 2 School of Animal Life Convergence Science, Institute of Genetic Engineering, Hankyong National University, Ansung, 17579 South Korea; 3 Department of Food Science and Nutrition, Hoseo University, Asan, 31499 South Korea

**Keywords:** R-eCG, CHO-S cells, MCR, Microarray, qRT-PCR, Immunohistochemistry

## Abstract

**Background:**

Equine chorionic gonadotropin (eCG) induces super-ovulation in laboratory animals. Notwithstanding its extensive usage, limited information is available regarding the differences between the in vivo effects of natural eCG (N-eCG) and recombinant eCG (R-eCG). This study aimed to investigate the gene expression profiles of mouse ovaries upon stimulation with N-eCG and R-eCG produced from CHO-suspension (CHO-S) cells. R-eCG gene was constructed and transfected into CHO-S cells and quantified. Subsequently, we determined the metabolic clearance rate (MCR) of N-eCG and R-eCG up to 24 h after intravenous administration through the mice tail vein and identified differentially expressed genes in both ovarian tissues, via quantitative real-time PCR (qRT-PCR) and immunohistochemistry (IHC).

**Results:**

R-eCG was markedly expressed initially after transfection and maintained until recovery on day 9. Glycan chains were substantially modified in R-eCG protein produced from CHO-S cells and eliminated through PNGase F treatment.

The MCR was higher for R-eCG than for N-eCG, and no significant difference was observed after 60 min. Notwithstanding their low concentrations, R-eCG and N-eCG were detected in the blood at 24 h post-injection. Microarray analysis of ovarian tissue revealed that 20 of 12,816 genes assessed therein were significantly up-regulated and 43 genes were down-regulated by > 2-fold in the group that received R-eCG (63 [0.49%] differentially regulated genes in total). The microarray results were concurrent with and hence validated by those of RT-PCR, qRT-PCR, and IHC analyses.

**Conclusions:**

The present results indicate that R-eCG can be adequately produced through a cell-based expression system through post-translational modification of eCG and can induce ovulation in vivo. These results provide novel insights into the molecular mechanisms underlying the up- or down-regulation of specific ovarian genes and the production of R-eCG with enhanced biological activity in vivo.

## Background

Equine chorionic gonadotropin CG (eCG) is a unique glycoprotein hormone because it has both luteinizing hormone (LH)- and follicle-stimulating hormone (FSH)-like biological activities [1, 2]. Glycoprotein hormones including LH, FSH, and thyroid-stimulating hormone (TSH) consist of non-covalently associated α- and β-subunits [3–6]. The α-subunit has an identical primary structure in the same species. However, each β-subunit is species-specific and structurally differs among species [7, 8].

The β-subunits of eCG and equine LH (eLH) have an identical primary structure and are reportedly expressed from the same gene [7, 9]. Thus, eCG is a potentially suitable good model to study structure-function relationships among gonadotropins owing to its dual LH- and FSH- activities in nonequid animals [10–12]. In equidaes, eCG exhibits only LH activity [12].

Owing to its long half-life in blood, a single dose of eCG, as opposed to multiple doses, is adequate to stimulate ovarian gene expression [13]. Furthermore, eCG and human CG (hCG) together stimulate ovulation in rats and mice [14, 15]. Moreover, eCG administration in cows is reportedly associated with an increase in their ovulation rate [16], particularly in early postpartum calves [17, 18]. Therefore, we speculate that ovulation rate is very important to determine the litter size in experimental animals and animal stock farms.

The glycosylation sites at amino acid residue 52 in the α-subunit of human FSH (hFSH) [19] and hCG [5] and residue 56 in eCG [7] are important for signal transduction when the cAMP response is impaired, and the binding activities of these hormones are increased by 2- to 3-fold [20], consistent with our previous findings [2, 6]. Thus, post-translational glycosylation of glycoprotein hormones plays a pivotal role in receptor-mediated signal transduction. N- and O-linked oligosaccharides at residue 56 of the α-subunit of eCG and a C-terminal extension (residues 114–149) in the β-subunit are included in vectors expressing eCG gene to produce recombinant-eCG (R-eCG) and to investigate the role of these regions in the biological activity of eCG.

High-throughput RNA sequencing and microarray analysis are useful during transcriptome profiling and gene expression analysis [21, 22]. A microarray contains thousands of millions of complementary DNA fragments or oligonucleotides that hybridize with specific RNA molecules in a sample [22]. A recent study revealed differentially expressed genes (DEGs) upon RNA-seq using ovarian tissue of dairy goats upon repeated eCG treatment [23], indicating that three-time eCG treatment dysregulated several ovarian genes including glucagon, follistatin-related protein 3 (FSTL3), and aquaporin-3 (AQP3), thereby reducing reproductive function.

We previously attempted to assess the different roles of R-eCG with respect to their attached oligosaccharides [2, 24], glycosylation sites for LH- and FSH-like activity [2], tethered R-eCGs [6], internalization of rat FSH and LH receptors by R-eCG [25], and signal transduction through eel FSH receptor and LH receptor by R-eCG and Natural-eCG (N-eCG) [26]. Furthermore, we analyzed the ovulation rates between N-eCG and deglycosylated R-eCGs in mice [27] and demonstrated that deglycosylated R-eCG mutants were induced at markedly lower levels in nonfunctional oocytes compared to N-eCG treated group. Non-functional oocytes in N-eCG and R-eCG mutants were approximately 20 and 2%, respectively. Numerous studies have reported the effects of a combination of eCG and hCG on reproductive performance and estrous synchronization [13–16]. However, no studies have investigated the effects of N-eCG and R-eCG on gene regulation through RNA-based microarray analysis.

In the present study, we hypothesized that treatment of ovarian tissues with N-eCG and R-eCG results in different DEG profiles. We produced R-eCG proteins in CHO-S cells, characterized their physiological function in vivo, and analyzed the differences in gene expression profiles through microarray analysis.

## Results

### Production of R-eCG and western blot analysis

**Fig. 1:**
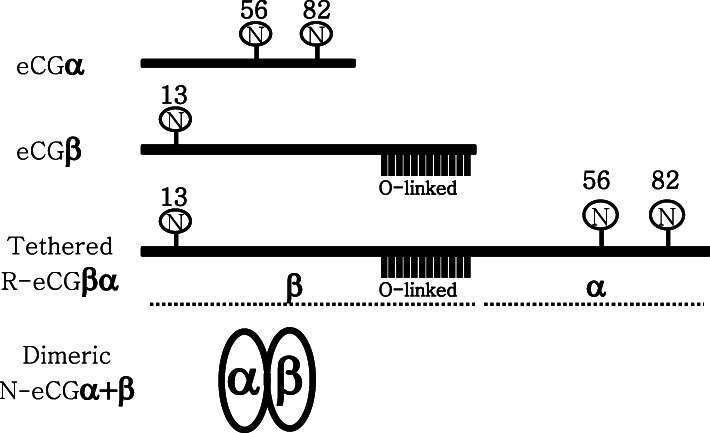
A schematic representation of tethered R-eCG. The wild-type protein with N- and O-linked oligosaccharide sites on eCG are shown. The circle “N” denotes an N-linked oligosaccharide. eCG α-subunit consists of 96-amino acids identical to those of LH, FSH, and TSH. eCG α-subunit contains two N-linked oligosaccharides at amino acid positions 56 and 82. eCG β-subunit contains one N-linked oligosaccharide site at position 13 and up to 12 O-linked oligosaccharide sites in the C-terminal region. Thus, tethered eCGβα gene was linked to C-terminal region of β-subunit with α-subunit without signal sequence. Natural eCG model is displayed as dimeric N-eCGα+β

eCG contains two N-linked glycosylation sites at amino acid positions 56 and 82 in the α-subunit of eCG. The β-subunit of eCG contains one N-linked glycosylation site at position 13 and approximately 12 O-linked glycosylation sites at the C-terminal region (Fig. 1). Thus, we constructed an expression vector encoding the tethered R-eCG mutant, which was linked with the C-terminal region of the β-subunit without the signal peptide region of the α-subunit composed of 24 amino acids. Tethered R-eCG gene is comprised of 813 bp containing signal sequence 60 bp of eCG β-subunit as shown in Fig. 1.

**Fig. 2:**
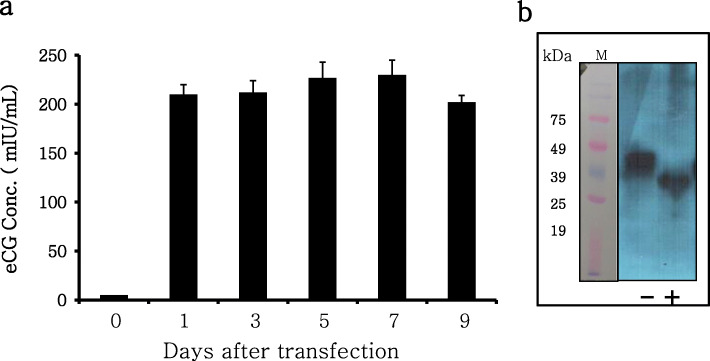
Quantitative analysis and western blot analysis of tethered R-eCG. **a** Quantities of *myc*-tagged R-eCG mutants were analyzed by an ELISA. The *myc*-tag (Glu-Gln-Lys-Leu-Ile-Ser-Glu-Glu-Asp-Leu) was inserted between the first and second amino acid residue of the β-subunit of mature eCG protein. **b** Western blot analysis of tethered R-eCG. The proteins in conditioned media were separated via SDS-PAGE and electro-transferred to a blotting membrane. The proteins were detected with antibodies against the *myc*-tag. Proteins for western blotting were also treated with N-Glycosidase F. -: not treated, +: treated with N-Glycosidase-F

R-eCG expression levels were markedly increased to 210 ± 10.3 mIU/mL on day 1 after transfection. These levels were consistently maintained until day 9, being 212 ± 12.7, 227 ± 16.1, 230 ± 15.6, and 202 ± 7.8 mIU/mL at 3, 5, 7, and 9 d, respectively (Fig. 2a). R-eCG was efficiently secreted into the cell culture medium. eCG levels markedly increased initially upon transfection and were maintained until recovery on day 9.

Further, we analyzed the molecular weight of R-eCG. On western blot analysis, the approximate molecular weight of R-eCG was 40–46 kDa (Fig. 2b). After deglycosylation with PNGase F, the molecular weight significantly decreased to approximately 30–36 kDa (Fig. 2b). The glycan chains were substantially modified post-translation in tethered R-eCG, confirming the loss of the its chains upon PNGase F treatment.

### Metabolic clearance rates (MCRs) of N-eCG and tethered R-eCG in vivo

**Fig. 3:**
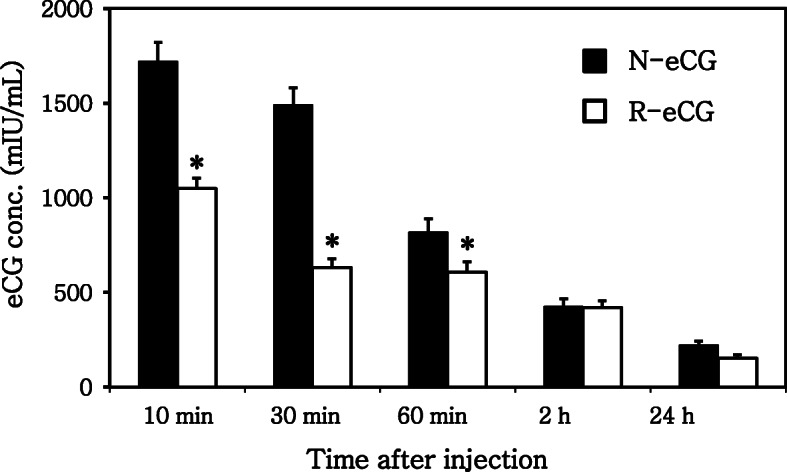
The metabolic clearance rate (MCR) of N-eCG and of R-eCGβ/α. Both eCGs were intravenously administrated at 5 IU through the tail vein. Blood samples were collected after 10 and 30 min and 1, 2, and 24 h. The samples were centrifuged at 5000 rpm for 15 min at 4 °C, and eCG concentrations in the serum were estimated using a PMSG ELISA kit. The levels of eCG were analyzed via sandwich ELISA in triplicate. Superscripts indicate significant differences in the groups (*p* < 0.05)

To analyze the MCR, eCG was detected in both groups (~ 550 mIU/mL) in the serum at 1 h after injection, as shown in Fig. 3. Although the MCR was slightly higher in the R-eCG–treated groups, no significant difference was observed between N-eCG and R-eCG treatment after 1 h. Their concentrations were low (~ 100 mIU/mL) until 24 h. These results indicate that R-eCG produced herein had a normal MCR and induced ovulation, as previously described [27].

### Comparison of ovarian gene expression profiles between groups treated with N-eCG and R-eCG

**Fig. 4:**
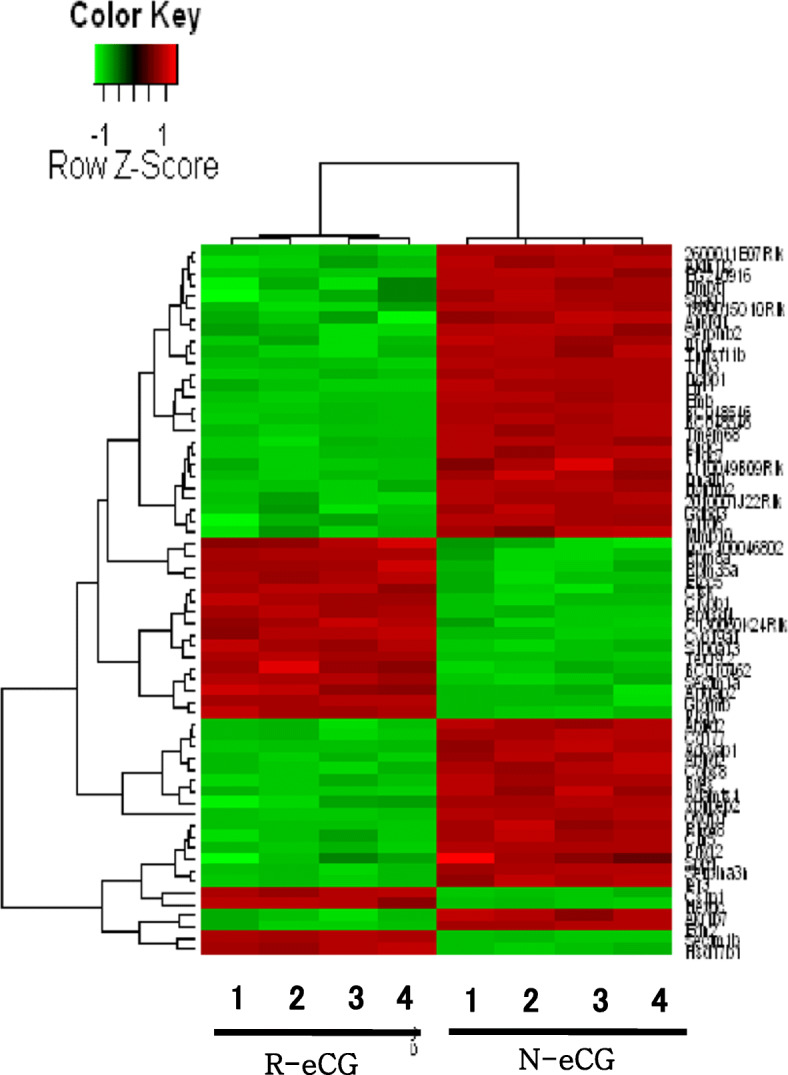
Hierarchical clustering of gene expression profiles in N-eCG-treated and R-eCG-treated ovarian tissues. The ovaries were excised from 8-week-old ICR female mice. The mice were induced to superovulate with 10 IU of N-eCG or R-eCG and then 10 IU of hCG after 48 h, and the ovulated oocytes were collected in an oviduct ampulla after 13 h. Thereafter, the ovaries were harvested after 13 h and RNA was analyzed via microarray analysis. Gene expression levels were evaluated through microarray analysis with 12,816 gene probes. Genes showing > 2-fold differences in expression were identified. The expression of 63 (0.49%) of 12,816 genes differed by at least 2-fold between N-eCG-treated and R-eCG-treated ovaries. Twenty of 63 genes were up-regulated in R-eCG-treated ovaries and 43 genes were down-regulated

Global gene expression profiles were analyzed in mouse ovarian tissue treated with N-eCG and R-eCG via microarray analysis. The ovarian tissues were harvested at 13 h upon combinational treatment (10 IU of eCG followed by 10 IU of hCG after 48 h). Gene expression levels were analyzed via microarray analysis with 12,816 gene probes. Genes showing a > 2-fold difference in expression levels were identified in eight ovaries (N-eCG: four, R-eCG: four). Figure 4 shows the differences in gene expression profiles between the two samples.

Expression profiles of 63 of 12,816 (0.49%) genes differed at least by 2-fold between the N-eCG and R-eCG groups (Fig. 4). Table 1 shows the significantly up-regulated genes (> 2-fold) in R-eCG-treated ovaries. Of the 20 (0.16%) genes up-regulated in the R-eCG group, six genes (*Ctsk*, *Crybb1*, *Rbm8a*, *Sectm1b*, *Tex19.2*, and *Rpusd4*) (30%) were up-regulated 3-fold. The number of genes down-regulated by > 2-fold in the R-eCG–treated ovaries was 43 (0.34%) (Table 2). Seven genes (*Ovgp1*, *BC048546a*, *BC048546b*, *Emb*, *Tmem68*, *Dcpp1*, and *Ltf*) were down-regulated (> 3-fold) among the 43 genes down-regulated in R-eCG-treated ovaries (Table 2). Only 63 (0.49%) genes were differentially expressed (> 2-fold) in R-eCG-treated ovaries compared to N-eCG treated ovaries.

**Table 1:** Genes that were up-regulated (fold-change) in R-eCGtreated ovaries

No.	Symbol	Accession No.	Fold R-eCG/N-eCG
1	Ercc5	NM_011729.1	2.04
2	Hexdc	NM_001001333.1	2.05
3	Arfgap2	NM_023854.1	2.16
4	Rbm35a	NM_194055.1	2.20
5	LOC100046802	XM_001476835.1	2.30
6	S100a13	NM_009113.3	2.46
**7**	**Hsd17b1**	**NM_010475.1**	**2.48**
8	Prcp	NM_028243.2	2.49
9	Cyp19a1	NM_007810.2	2.50
10	BC010462	NM_145373.1	2.62
**11**	**Sectm1a**	**NM_145373.2**	**2.64**
**12**	**Gpnmb**	**NM_053110.3**	**2.82**
13	Csrp1	NM_007791.4	2.88
14	C130060K24Rik	NM_175524.3	2.97
**15**	**Ctsk**	**NM_007802.3**	**3.11**
16	Crybb1	NM_023695.2	3.17
17	Rbm8a	NM_025875.1	3.22
**18**	**Sectm1b**	**NM_026907.3**	**3.47**
**19**	**Tex19.2**	**NM_027622.2**	**3.57**
20	Rpusd4	NM_028040.2	3.80

Bold genes were adjusted to RT-PCR and qRT-PCR

**Table 2:** Genes that were down-regulated (fold-change) in R-eCG-treated ovaries

No.	Symbol	Accession No.	Fold R-eCG/N-eCG
**1**	**Ovgp1**	**NM_007696.2**	**-18.31**
**2**	**BC048546**	**NM_001001179.2**	**-10.61**
3	BC048546	NM_001001179.1	-10.28
4	Emb	NM_010330.3	-6.64
**5**	**Tmem68**	**NM_028097.3**	**-5.81**
**6**	**Dcpp1**	**NM_019910.2**	**-5.10**
7	Ltf	NM_008522.3	-4.97
8	1500015O10Rik	NM_024283.2	-3.09
9	Dynlrb2	NM_029297.1	-2.97
10	Il1rn	NM_031167.3	-2.89
**11**	**Prkg2**	**NM_008926.3**	**-2.81**
12	Mmp10	NM_019471.2	-2.78
13	Spag1	NM_012031.1	-2.77
**14**	**Edn2**	**NM_007902.2**	**-2.73**
15	Tnfrsf11b	NM_008764.3	-2.62
16	Cops8	NM_133805.3	-2.55
17	Trib3	NM_175093.2	-2.53
18	EG240916	NM_177723.2	-2.51
19	Cln5	XM_127882.3	-2.49
20	Bves	NM_024285	-2.46
21	Spp1	NM_009263.1	-2.43
22	Adcyap1	NM_009625.2	-2.36
23	Dmbt1	NM_007769.1	-2.35
24	Fndc7	NM_177091.2	-2.33
25	Rhox8	NM_001004193.2	-2.32
26	2600011E07Rik	NM_028113.1	-2.24
**27**	**Adamts1**	**NM_009621.3**	**-2.24**
28	Serpinb2	NM_011111.3	-2.21
29	Dnali1	NM_175223.2	-2.20
30	Abhd2	NM_018811.6	-2.19
31	Kndc1	NM_177261.4	-2.19
32	Xpnpep2	NM_133213.2	-2.16
33	Abhd2	NM_018811.6	-2.15
34	Ier3	NM_133662.2	-2.13
35	Ankrd1	NM_013468.2	-2.12
**36**	**Akr1b7**	**NM_009731.1**	**-2.09**
37	Serpina3n	NM_009252.2	-2.09
38	V1rd6	NM_030738.1	-2.08
39	Gdpd3	NM_024228.2	-2.08
40	1110049B09Rik	NM_001024478.1	-2.05
41	Aldh1l2	NM_153543.1	-2.02
42	Cd177	NM_026862.3	-2.02
43	2010001J22Rik	NM_001013022.1	-2.00

Bold genes were adjusted to RT-PCR and qRT-PCR

Further, we analyzed the data to gain insight into the biological processes and functions of the DEGs. The distribution of the 63 DEGs (at least 2-fold) between ovaries treated with N- eCG and R-eCG and their distribution in different Gene Ontology (GO) categories were analyzed (Supplementary Material Fig. 1). GO analysis was performed using the Panther database (http://www.pantherdb.org). GO terms “biological process” were the most represented (> 4 genes) among R-eCG-treated ovarian tissue, including “signal transduction (16 genes),” “developmental processes (14),” “protein metabolism and modification (9),” “cell culture and motility (5),” and “nucleoside, nucleotide and nucleic acid metabolism (4).” In category “molecular function,” these genes were classified into 18 subcategories through GO analysis, with the largest number of genes represented in “protease (6 genes),” “signaling molecule (5),” “oxidoreductase (5),” “nucleic acid binding (5),” and “hydrolase (5).” Seven genes were categorized as “molecular function unclassified” (Supplementary Material Fig. 2). The number of classified genes is the number of genes in categories after excluding overlapping categories.

### Gene expression analysis through quantitative reverse-transcription PCR (qRT-PCR) analysis

**Fig. 5:**
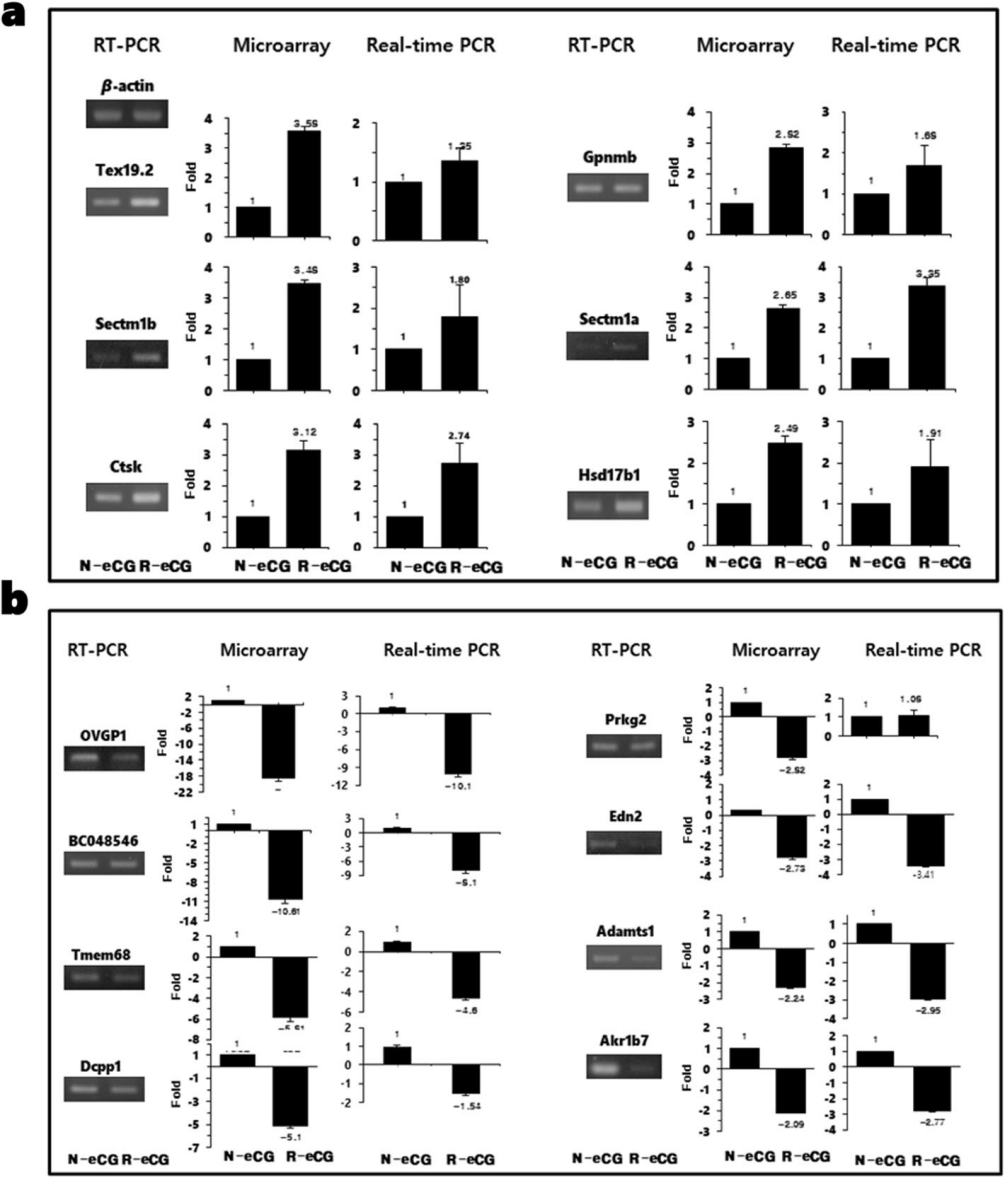
Quantitative real-time PCR (qRT-PCR) and reverse-transcription PCR (RT-PCR) analyses. **a** Fourteen genes from different categories were analyzed via qRT-PCR. Six genes were up-regulated in the R-eCG-treated ovaries. **b** The other eight genes were down-regulated in R-eCG-treated ovaries. The microarray results were compared and further analyzed via RT-PCR and qRT-PCR. *Actb* served as an endogenous control

To validate the results of microarray analysis, we performed RT-PCR and qRT-PCR analyses using specific primers (Supplementary Material Table 1) for the 14 genes identified herein (Fig. 5a, b). Among the up-regulated genes identified through microarray analysis of R-eCG-treated ovaries, six genes, i.e., *Tex19.2*, *Sectm1b*, *Ctsk*, *Gpnmb*, *Sectm1a*, and *Hsd17b1,* were confirmed to be up-regulated through qRT-PCR (Fig. 5a). Among the down-regulated genes, eight genes, i.e., *OVGP1*, *BC048546*, *Tmem68*, *Dcpp1*, *Prkg2*, *Edn2*, *Adamts1*, and *Akr1b7,* were confirmed to be down-regulated by > 2-fold in the R-eCG-treated mouse ovarian tissue, of which seven, i.e., *OVGP1*, *BC048546*, *Tmem68, Dcpp1*, *Edn2*, *Adamts1*, and *Akr1b7*, were confirmed to be down-regulated via qRT-PCR analysis (Fig. 5b). Nonetheless, one gene, *Prkg2,* displayed no significant change in expression levels upon qRT-PCR analysis. The fold-change in the expression levels of these genes was consistent with the results of the microarray analysis, confirming that the results of qRT-PCR analysis correlated with those of the microarray analysis.

### Immunohistochemical analysis of ovarian tissue

**Fig. 6:**
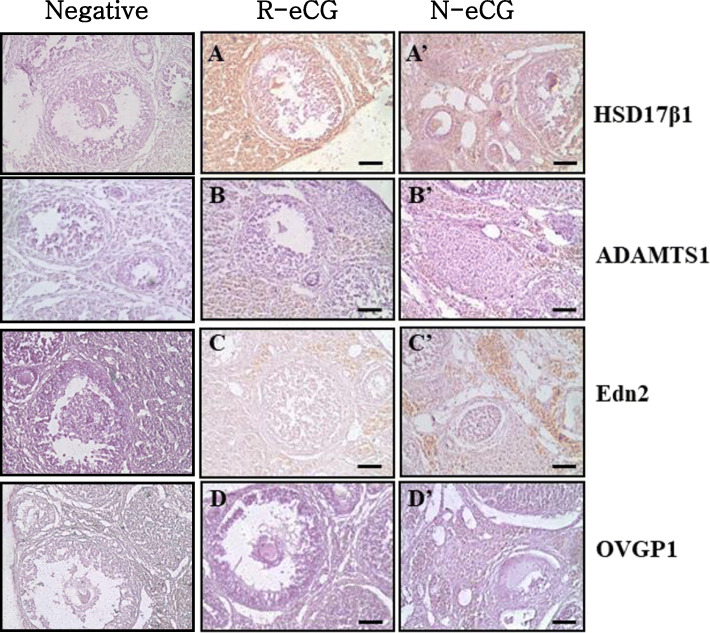
Localization of HSD17β1, ADAMTS1, EDN2, and OVGP1. The ovaries were induced to superovulate with 10 IU of either N-eCG or R-eCGβ/α, followed by 10 IU of hCG after 48 h. Representative immunohistochemical analyses for HSD17β1, ADAMTS1, EDN2, and OVGP1 were conducted with antisera, and a goat anti-rabbit IgG antibody (secondary antibody). According to the microarray and qRT-PCR results, HSD17β1 was up-regulated in the R-eCG-treated ovaries, while the other three proteins (ADAMTS1, EDN2, and OVGP1) were up-regulated in the N-eCG-treated ovaries. Immunohistochemistry was performed with a Vectastain ABC kit. Scale bar = 200 μm

To determine the cell types expressing four proteins (HSD17β1, ADAMTS1, Edn2, and OVGP1), immunohistochemical analysis was performed for the same ovarian tissues used for microarray analysis (Fig. 6). Among the up-regulated genes in the R-eCG-treated ovarian tissue, HSD17β1 was localized in the granulosa cells and theca folliculi. Among the down-regulated genes in R-eCG-treated ovarian tissue, ADAMTS1, which is required for normal ovulation and is localized in the cumulus oocyte complex during the preovulatory stage, was also localized in granulosa cells. Edn2, which is transiently expressed in granulosa cells immediately prior to ovulatory follicle rupture, was also strongly expressed in the ovarian stroma of a N-eCG-treated ovarian tissue. OVGP1, which improves the efficiency of in vitro fertilization and increases the number of fertilized eggs, was weakly expressed in the ovaries after ovulation. These results indicate that the expression of these four proteins was directly correlated with the time of ovulation in mice.

## Discussion

This study examined the biological activity of tethered R-eCG, containing N- and the O-linked oligosaccharide chains and their MCR in vivo. Furthermore, this study evaluated differential gene expression profiles in mouse ovaries stimulated with N-eCG and R-eCG in combination with hCG. The present study shows differences in the up- and down-regulated genes (> 2-fold) in ovarian tissues treated with N-eCG and R-eCG.

Thus, far, we have expressed R-eCG in only CHO-K1 cells and stable CHO-K1 cells under G418 selection [6, 25–27]. Hence, levels of secreted R-eCG at 24 h post-transfection have remained unknown. However, supernatants of the culture media of CHO-S cells were recovered until 9 days after transfection. In the present study, single-chain R-eCG was markedly up-regulated on day 1 after transfection in CHO-S cells. However, R-eCG with a C-terminal deletion in the β-subunit was detected at a low concentration on day 1 and 3 post-transfection (data now shown). The present results indicate that the CTP region including up to 12 O-linked oligosaccharides plays a pivotal role in the early secretion of eCG from cells into the supernatant medium after transfection.

Various studies have reported that R-eCG proteins lead to the production and secretion of stable heterodimeric eCG in COS-7 cells [28] and infected Sf9 cells [29], with thermal stability similar to that of native pituitary LH [30]. Secreted single-chain eCG in COS-7 cells is detectable as a doublet of 46 and 44 kDa [12]. The present results show that the molecular weight of R-eCG greatly decreases upon elimination of the N-linked oligosaccharide chains via PNGase F treatment, decreasing the molecular weight to approximately 30–36 kDa. Our results are consistent with those of other studies, suggesting that R-eCG contained highly modified N-linked glycosylation sites in COS-7 cells and CHO-K1 cells post-translation [12, 27].

Furthermore, R-eCG mutants deglycosylated through site-directed mutagenesis was markedly low in number (< 2.4%) in nonfunctional oocytes in comparison with N-eCG (21.2%) [27]. These results suggest a specific model for ovulation without displaying a long-half-life and to only induce functional oocytes in experimental animals despite using N-eCG. Furthermore, the MCR of R-eCG was somewhat higher than that of N-eCG at 10–60 min after injection and was similarly maintained at 2 and 24 h. These MCR results suggest that R-eCG derivatives can be beneficially utilized for animal experiments. In the present study, the expression level of R-eCG produced from CHO-S cells was extremely low for experimental animals and animal stock farms application. Thus, plans to isolate the single colony cells expressing lots of R-eCG quantity using the DG44 cells which would produce more R-eCG are underway.

Furthermore, we previously reported that R-eCG exerts dual LH- and FSH-like activity in in vitro bioassays involving rat Leydig and granulosa cells, respectively [2, 6]. Moreover, we previously reported that R-eCG has both LH- and FSH-like activity in cells expressing rat LH/CGR and rat FSHR [25]. Nevertheless, no studies have examined differential gene expression in ovaries stimulated with N-eCG and R-eCG. We performed gene expression profiling for ovarian tissue through microarray analysis after administration of N-eCG or R-eCG. We identified genes up- and down-regulated by > 2-fold. The present results show that 63 genes were up- and down-regulated (0.49% of 12,816 genes in R-eCG-injected ovaries). These changes in gene expression profiles directly render oocytes nonfunctional upon comparing N-eCG-treated and R-eCG-treated ovaries, suggesting that tethered R-eCG derivatives used herein can cause slightly aberrant gene expression in the ovaries and produce functional oocytes without nonfunctional oocytes, in comparison with N-eCG-treated ovaries. We also reported the ovulation rate, indicating that deglycosylated R-eCG mutants displayed only 2% nonfunctional oocytes compared to about 20% in N-eCG treated ovaries [27]. We revealed that gene expression profiles differ slightly differ between N-eCG and R-eCG mutants, thus suggesting that the glycan chains play a pivotal role in the ovulation rate and gene expression of ovaries treated with R-eCG compared to N-eCG treated ovaries.

In the “biological process” category, the largest number of deregulated genes included signal transduction (16 proteins; Supplementary Material Figs. 1 and 2). In contrast, the largest number of genes (6 genes) among the 18 “molecular function” categories were present in “proteases.” Seven genes were categorized as “molecular function unclassified.” We assessed differences in the expression of ovary-specific genes between groups treated with N-eCG and R-eCG through qRT-PCR analysis. The differences in gene expression were confirmed for six genes. These genes, *Tex19.2*, *Sectm1b*, *Ctsk*, *Gpnmb*, *Sectm1a*, and *Hsd17β1*, were specifically over-expressed in R-eCG-treated ovaries. Among the genes found to be down-regulated on microarray analysis, seven genes were confirmed to be down-regulated through qRT-PCR analysis. These differences should be further assessed through a systematic study.

Immunohistochemical analysis was conducted to determine the cell type responsible for protein expression in the ovaries. We first confirmed that 17β-hydroxysteroid dehydrogenase type 1 (17β-HSD1), which catalyzes the conversion of estrone to estradiol, is primarily localized in ovarian granulosa cells after R-eCG injection. Our results are consistent with those of another study, showing that 17β-HSD1 is expressed in the placenta and ovarian granulosa cells [31]. Some studies have reported that ADAMTS-1 is induced in granulosa cells in preovulatory follicles after LH administration [32] and is important for follicular development and the maintenance of normal granulosa cell layers in follicles [33]. Endothelin-2 (Edn2), a potent vasoconstrictive peptide, is abundantly produced by preovulatory follicles during ovulation at the onset of CL formation [34]. Edn2 directly induces vascular endothelial growth factor in granulosa cells of the bovine ovary [35] and ovulation and CL formation are significantly impaired in Edn2-knockout mice [34]. Oviduct-specific glycoprotein (OVGP1), also known as oviductin, is the major non-serum glycoprotein in the oviduct fluid during fertilization and increases the number of fertilized eggs and promotes early embryonic development [36]. Furthermore, Edn2 and OVGP1 are primarily localized in the ovaries after N-eCG administration. These results suggest that 17β-HSD1, ADAMTS-1, Edn2, and OVGP1 perform pivotal functions as ovulatory factors during ovulation in mice. Although the differences in MCR and gene expression profiles were expected, the glycosylated glycans were the absolutely cause of these changes, indicating that R-eCG produced from CHO cells is highly modified with mannose, with small amount of sialic acid attached to the end of glycosylated chains. Therefore, mammalian cells should be developed to investigate the role of modified glycosylation on the expression levels of R-eCG.

## Conclusions

This study shows that R-eCG produced from CHO-S cells has high biological activity in vivo. Although R-eCG disappears rapidly from the circulation immediately after its administration, R-eCG displayed a wide range of biological activity including the induction of ovulation and oogenesis. We showed that 63 ovarian genes were differentially expressed between N-eCG-treated and R-eCG-treated ovaries. Differential expression patterns of these genes were further confirmed through RT-PCR, qRT-PCR, and immunostaining analyses. Further systematic analyses are required to investigate the role of these DEGs in ovulation. Nevertheless, our results suggest that these differences may have resulted from the nature of the hormone, including oligosaccharides and folding. Therefore, R-eCG derivatives can potentially be produced at high levels with high biological activity to induce oocytes in vivo.

## Methods

### Materials

The oligonucleotides used herein were synthesized by Genotech (Daejon, Korea). The restriction enzymes and the DNA ligation kit were purchased from Takara (Tokyo, Japan). The QIAprep-Spin plasmid kit was acquired from QIAGEN, Inc. (Hilden, Germany). The Lumi-Light western blot kit was purchased from Roche (Basel, Switzerland), and the pcDNA3 mammalian expression vector, FreeStyle CHO-S suspension cells, PNGase F, FreeStyle MAX transfection reagent, and TRIzol reagent were obtained from Invitrogen (Carlsbad, CA, USA). The PMSG ELISA kit was purchased from DRG International, Inc. (Mountain side, NJ, USA), Centriplus Centrifugal Filter Devices from Amicon Bio separations (Merck, Billerica, MA, USA), and an anti-*myc* antibody and antibodies against HSD17β1, ADAMTS1, Edn2, and OVGP1 were purchased from Santa Cruz Biotechnology (Dallas, TX, USA). Disposable spinner flasks were obtained from Corning Inc. (Corning, NY, USA). A peroxidase-conjugated anti-mouse IgG antibody was obtained from Bio-Rad (Hercules, CA, USA), whereas pregnant-mare serum gonadotropin (eCG; ≥1000 IU/mg, G4877) and hCG (5000 IU, CG5) from Sigma-Aldrich Corp. (St. Louis, MO, USA), as were all other reagents. PMSG and hCG reagents are generally used to induce the ovulation in mice as we have previously reported [15]. All protocols complied with the approved Guidelines for Animal Experiments of Hankyong National University, Korea, and were approved by the Animal Care and Use Committee of Hankyong National University, Korea (Approval ID: 2015–8).

### Construction of tethered eCG gene

cDNA encoding the tethered R-eCGβ/α was inserted into the mammalian expression vector pcDNA3, as previously reported [6]. The same method was used to insert a *myc* tag (Glu-Gln-Lys-Leu-Ile-Ser-Glu-Glu-Asp-Leu) between the first and second amino acid residues of the β-subunit of the mature eCG protein [27]. Plasmid DNA was then purified and sequenced in both directions through automated DNA sequencing to ensure correct inserts. The cloned expression vector of tethered eCG was designated as pcDNA3-eCGβ/α, as previously reported [6]. A schematic representation for tethered R-eCG β/α is shown in Fig. 1.

### Cell culture and generation of tethered R-eCG

In CHO-S cells, the tethered R-eCG expression vector was transfected into CHO-S cells using the FreeStyle MAX reagent (Invitrogen; Carlsbad, CA, USA) transfection method, in accordance with manufacturer’s instructions. Flasks were placed on an orbital shaking platform, rotating at 120–135 rpm at 37 °C in a humidified atmosphere of 8% CO_2_ in air. On transfection, the cell density was approximately 1.2–1.5 × 10^6^ cells/mL. The plasmid DNA (260 μg) and a FreeStyle™ MAX Reagent complexes were gradually added to 200 mL of medium containing cells. Finally, culture media were sampled on day 9 after transfection and centrifuged to eliminate cell debris. The supernatant was sampled and stored at − 20 °C until the assay. The samples were concentrated using a Centricon filter or by freeze-drying and mixed with PBS.

### Quantification of R-eCG proteins

R-eCG protein was quantified with the PMSG ELISA kit (DRG Diagnostics; Mountain side, NJ, USA). Briefly, the PMSG standard and R-eCG samples (100 μL) were dispensed into the wells of a plate coated with the antibody and incubated for 60 min at ambient temperature. After rinsing thrice, 100 μL of anti-PMSG antibody conjugated with horseradish peroxidase was added into each well and incubated for 60 min. The plate wells were rinsed five times, and substrate solution (100 μL) was added and incubated for 30 min at ambient temperature. Finally, 50 μL of a stop solution was added and the absorbance was measured at 450 nm, using a microtiter plate reader Cytation™ 3 (BioTeK, Winooski, VT, USA). The average absorbance of each standard was plotted against its corresponding concentration in a linear–log graph. We determined the average absorbance of each sample to determine the corresponding PMSG value via simple interpolation through a standard curve. Given the low expression level of R-eCG in CHO-S cells, samples were concentrated approximately 40 ~ 50 times for application of R-eCG in MCR and superovulation. Concentrated R-eCG samples were diluted about 40 times for standard curve calibration. Samples for the standard curve were 0. 25, 100, 200, 400, 800 mIU/mL. Finally, 1 IU was considered 100 ng in accordance with the conversion factor of the suggested assay protocol.

### Detection of R-eCGs via western blotting and enzymatic digestion of N-linked oligosaccharides

Concentrated sample media were subjected to SDS-PAGE (12.5% resolving gel) via the Laemmli method [37]. After SDS-PAGE, the proteins were electro-transferred to a nitrocellulose membrane for 2 h in a Mini Trans-Blot Electrophoretic Transfer cell. To eliminate all N-linked oligosaccharides, the R-eCG sample was incubated for 24 h at 37 °C with PNGase F [2 μL of the enzyme (2.5 U/mL) per 30 μL of sample+ 8 μL of 5× reaction buffer]. The reaction was terminated by boiling for 10 min, and the samples were subjected to SDS-PAGE and the proteins were electro-transferred on to a membrane. After blocking the membrane with a 1% blocking reagent for 1 h, followed by probing with monoclonal anti-*myc* antibody (1: 5000) for 2 h, the membrane was washed and probed with a secondary antibody (peroxidase-conjugated anti-mouse IgG antibody 37.5 μL/15 mL of the blocking solution) for 30 min. The membrane was then incubated for 5 min with 2 mL of the Lumi-Light substrate solution and X- ray film was exposed to the membrane for 1–10 min.

### Assessment of the MCR of N-eCG and R-eCG

Each animal was intravenously administered 5 IU of N-eCG or R-eCG through the tail vein to determine the 50% dose for the induction of superovulation. Blood was sampled from the transorbital vein in heparinized microhematocrit tubes. Blood samples were obtained at 10 and 30 min and at 1, 2, and 24 h and centrifuged for 15 min at 5000 rpm at 4 °C, and plasma eCG concentrations were estimated using the PMSG ELISA kit (DRG Diagnostics).

### Animals

The MCRs of N-eCG and R-eCG were determined in 8-week-old male B6D2F1 (C57BL6 × DBA/2) 12 mice. The female 16 mice (8-week-old B6D2F1; Oriental Bio, Gyeonggi, Korea) were superovulated by injection of 10 IU of N-eCG and R-eCG and then 10 IU hCG after 48 h. The ovarian tissues were sampled at 13 h after hCG administration. All mice were euthanized with carbon dioxide inhalation, and the ovarian tissues were collected at the end of study. All the mice were raised in an environment with the temperature of 23 ± 1 °C with regular 12 h light/dark cycle and allowed free access to feed and water. The animals were processed according to the Animal Care and Use Committee procedure. The protocol was approved by the Committee on Ethics of Animal Experiments at the Hankyong National University (Approval ID: 2015–8).

### Microarray analysis

Total RNA was extracted from ovaries, using TRIzol reagent, and purified using RNeasy columns in accordance with the manufacturers’ protocols, as previously described [15].

1. Labeling and purification

Total RNA was amplified and purified using an Ambion Illumina RNA amplification kit (Ambion, Austin, TX, USA) in accordance with the manufacturer’s instructions to obtain biotinylated cRNA. Briefly, 550 ng of total RNA was reverse-transcribed into cDNA with a T7 oligo(dT) primer. Second-strand cDNA was synthesized, transcribed in vitro, and labeled with biotin-NTP.

2) Hybridization and data export

Labeled cRNA samples (0.75 μg) were hybridized to the Illumina MouseRef-8 v2 expression BeadChip (Illumina, Inc., San Diego, CA, USA) for 16–18 h at 58 °C. Array signals were detected using Amersham Fluorolink Streptavidin-Cy3 (GE Healthcare Bio-Sciences, Little Chalfont, UK) in accordance with the manufacturer’s instructions. Arrays were scanned using an Illumina bead array reader (confocal scanner). Array data were analyzed using in Illumina Genome Studio v.2009.2 software (Gene Expression Module v.1.5.4).

3) Raw data preparation and statistical analysis

Raw data were extracted using the software provided by the manufacturer (Illumina Genome Studio v.2009.2) and filtered using a detection *p*-value of < 0.05 (a signal value higher than that of the background was necessary to set the detection *p*-value of < 0.05). The selected gene signal value was logarithmically transformed and normalized to XYZ. Comparative analysis between two groups was conducted on the basis of the *p*-value evaluation, via the local-pooled-error test (adjusted Benjamini–Hochberg false discovery rate had to be< 5%) and the fold-change. Biological ontology-based analysis was performed for the Panther database (http://www.pantherdb.org). Furthermore, genes whose expression levels differed by > 2-fold were considered differentially expressed between the two groups.

### RT-PCR and qRT-PCR analyses

To validate the microarray data, 14 genes (up-regulated: *Tex19.2*, *Sectm1b*, *Ctsk*, *Gpnmb*, *Sectm1a*, and *Hsd17β1*; down-regulated: *OVGP1*, *BC048546*, *Tmem68, Dcpp1*, *Prkg2*, *Edn2*, *Adamts1*, and *Akr1b7*) from different groups were evaluated through RT-PCR and qRT-PCR analysis, their expression levels differed by > 2-fold. RT-PCR and qRT-PCR analysis was performed for the same ovarian tissue subjected to microarray analyses. Primer sequences are outlined in Supplementary Material Table 1 along with the primer annealing temperatures. The primers were designed using Primer3 software (http://www.bioneer.co.kr/tools/). Ovarian gene expression levels were then normalized to those of *Actb* via the 2^-ΔΔCT^ method for quantitative relation.

### Immunohistochemistry

Immunohistochemical staining of ovarian samples was performed using the Vectastain ABC kit (Vector Laboratories, Burlingame, CA, USA) in accordance with the manufacturer’s instructions. The samples were fixed in 10% neutral-buffered formalin at ambient temperature for 24 h and washed with PBS. Thereafter, the fixed samples were rehydrated in graded ethanol (EtOH) solutions (3 min each in 100% 2×; 95% 1×; 70% 1×; and 50% 1×) and embedded in paraffin. Paraffin-embedded tissues were sectioned into 8-μm-thick sections, which were then mounted onto poly-l-lysine-coated slides. The slides were boiled in 10 mM sodium citrate for 10 min and chilled on ice for 20 min. Thereafter, they were washed with 3% hydrogen peroxide for 10 min and blocked for 1 h at ambient temperature. The slides were incubated with the primary antibody and then with an anti-rabbit IgG antibody (secondary antibody). Finally, the slides were immunostained using the ABC detection kit in accordance with the manufacturer’s instructions and stained with DAB. The slides were examined under a Nikon Eclipse TE-2000-E confocal microscope (Tokyo, Japan).

### Data and statistical analysis

Data are presented as mean ± SEM values. One-way ANOVA with Tukey’s multiple-comparison test was conducted to compare the results between samples. In figures, the superscripts indicate significant differences between groups (*p* < 0.05).

## Supplementary Information

**Additional file 1: Table S1**. List of primers used for RT-PCR and qRT-PCR. Fourteen genes from different categories were chosen for RT-PCR and qRT-PCR analyses. The gene for *β-*actin was used as the endogenous control. **Figure S1**. Gene ontology of biological processes and molecular functions. Genes distribution of > 2-fold differentially expressed genes between N-eCG and R-eCG. **Figure S2**. Gene ontology of biological processes and molecular functions. Gene ontology pie diagram of > 2-fold differentially expressed genes between N-eCG and R-eCG-treated ovaries. The up-regulated or down-regulated genes are categorized by the GO term “biological process and molecular function”.

**Additional file 2.**

**Additional file 3.**

## Data Availability

The datasets used and analyzed in the current study are available from the corresponding author on reasonable request.

## Abbreviations

R-eCG: Recombinant equine chorionic gonadotropin
N-eCG: Natural eCG
LH: Luteinizing hormone
FSH: Follicle-stimulating hormone
TSH: Thyroid-stimulating hormone
FSHR: FSH receptor
PMSG: Pregnant mare serum gonadotropin
MCR: Metabolic clearance rate
qRT-PCR: quantitative reverse-transcription-polymerase chain reaction
HSD: Hydroxysteroid dehydrogenase
SDS-PAGE: Sodium dodecyl sulfate-polyacrylamide gel electrophoresis
Edn2: Endothelin-2
OPGP1: Oviduct-specific glycoprotein1
