# Supplementary material for: Comparative gene expression profiling of mouse ovaries upon stimulation with natural equine chorionic gonadotropin (N-eCG) and tethered recombinant-eCG (R-eCG)
Source: BMC Biotechnol. 2020 Nov 11;20:59. doi: 10.1186/s12896-020-00653-8 (PMC7661263; doi:10.1186/s12896-020-00653-8)
Supplement: Supplementary file 1 — Additional file 1: Table S1. List of primers used for RT-PCR and qRT-PCR. Fourteen genes from different categories were chosen for RT-PCR and qRT-PCR analyses. The gene for β-actin was used as the endogenous control. Figure S1. Gene ontology of biological processes and molecular functions. Genes distribution of > 2-fold differentially expressed genes between N-eCG and R-eCG. Figure S2. Gene ontology of biological processes and molecular functions. Gene ontology pie diagram of > 2-fold differentially expressed genes between N-eCG and R-eCG-treated ovaries. The up-regulated or down-regulated genes are categorized by the GO term “biological process and molecular function”. [file 12896_2020_653_MOESM1_ESM.ppt]

## Slide 1
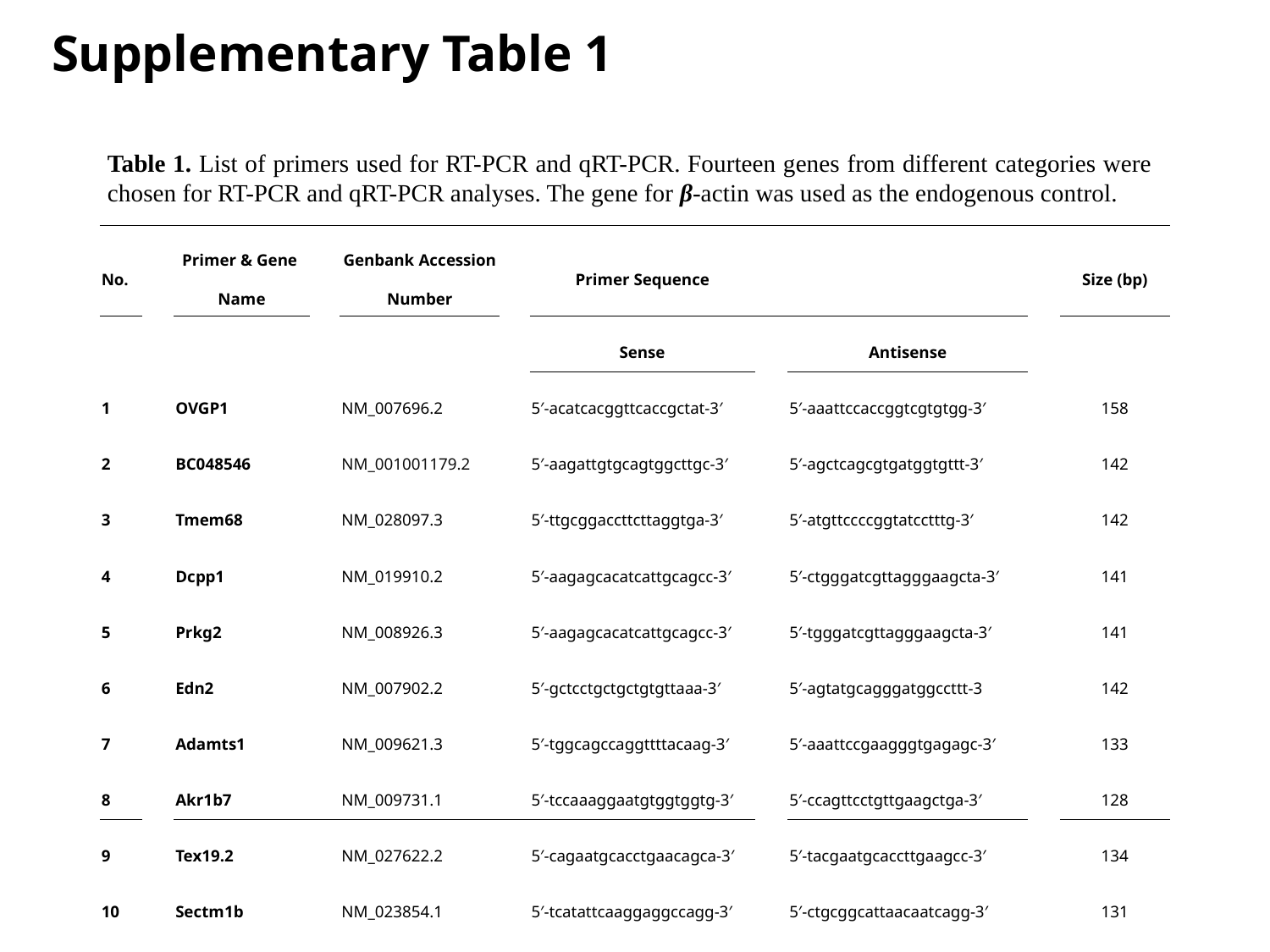

Supplementary Table 1
Table 1. List of primers used for RT-PCR and qRT-PCR. Fourteen genes from different categories were chosen for RT-PCR and qRT-PCR analyses. The gene for β-actin was used as the endogenous control.
| No. | | Primer & Gene Name | | Genbank Accession Number | | Primer Sequence | | | | Size (bp) |
| --- | --- | --- | --- | --- | --- | --- | --- | --- | --- | --- |
| | | | | | | Sense | | Antisense | | |
| 1 | | OVGP1 | | NM\_007696.2 | | 5′-acatcacggttcaccgctat-3′ | | 5′-aaattccaccggtcgtgtgg-3′ | | 158 |
| 2 | | BC048546 | | NM\_001001179.2 | | 5′-aagattgtgcagtggcttgc-3′ | | 5′-agctcagcgtgatggtgttt-3′ | | 142 |
| 3 | | Tmem68 | | NM\_028097.3 | | 5′-ttgcggaccttcttaggtga-3′ | | 5′-atgttccccggtatcctttg-3′ | | 142 |
| 4 | | Dcpp1 | | NM\_019910.2 | | 5′-aagagcacatcattgcagcc-3′ | | 5′-ctgggatcgttagggaagcta-3′ | | 141 |
| 5 | | Prkg2 | | NM\_008926.3 | | 5′-aagagcacatcattgcagcc-3′ | | 5′-tgggatcgttagggaagcta-3′ | | 141 |
| 6 | | Edn2 | | NM\_007902.2 | | 5′-gctcctgctgctgtgttaaa-3′ | | 5′-agtatgcagggatggccttt-3 | | 142 |
| 7 | | Adamts1 | | NM\_009621.3 | | 5′-tggcagccaggttttacaag-3′ | | 5′-aaattccgaagggtgagagc-3′ | | 133 |
| 8 | | Akr1b7 | | NM\_009731.1 | | 5′-tccaaaggaatgtggtggtg-3′ | | 5′-ccagttcctgttgaagctga-3′ | | 128 |
| 9 | | Tex19.2 | | NM\_027622.2 | | 5′-cagaatgcacctgaacagca-3′ | | 5′-tacgaatgcaccttgaagcc-3′ | | 134 |
| 10 | | Sectm1b | | NM\_023854.1 | | 5′-tcatattcaaggaggccagg-3′ | | 5′-ctgcggcattaacaatcagg-3′ | | 131 |
| 11 | | Ctsk | | NM\_194055.1 | | 5′-caagcttggcatctttccag-3′ | | 5′-atccagtgcttgcttccctt-3′ | | 127 |
| 12 | | Gpnmb | | XM\_001476835.1 | | 5′-tcaacgactctgccatttcc-3′ | | 5′-tttgcacggtgaggttaagg-3′ | | 129 |
| 13 | | Sectm1a | | NM\_009113.3 | | 5′-ctgtctgccaatggaaagga-3′ | | 5′-tcgtcctgggtgtctttgat-3′ | | 128 |
| 14 | | Hsd17b1 | | NM\_010475.1 | | 5′-tcgaaggtttgtgcgagagt-3′ | | 5′-gcccaccagcttttcataga-3′ | | 110 |
| | | β-actin | | - | | 5′-agagggaaatcgtgcgtgac-3 | | 5′-caatagtgatgacctggccgt-3′ | | 138 |

## Slide 2
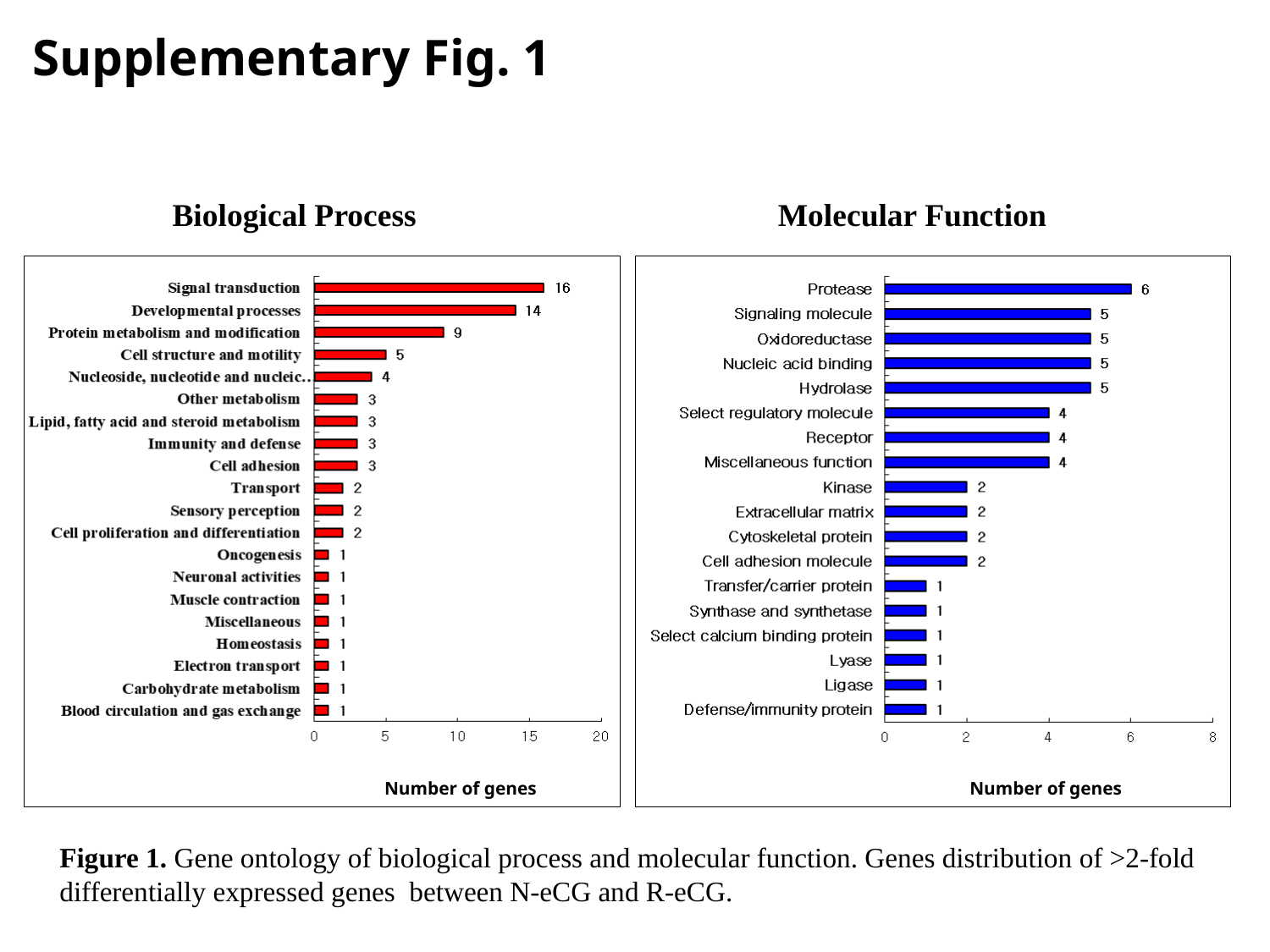

Supplementary Fig. 1
Biological Process
Molecular Function
Number of genes
Number of genes
Figure 1. Gene ontology of biological process and molecular function. Genes distribution of >2-fold
differentially expressed genes between N-eCG and R-eCG.

## Slide 3
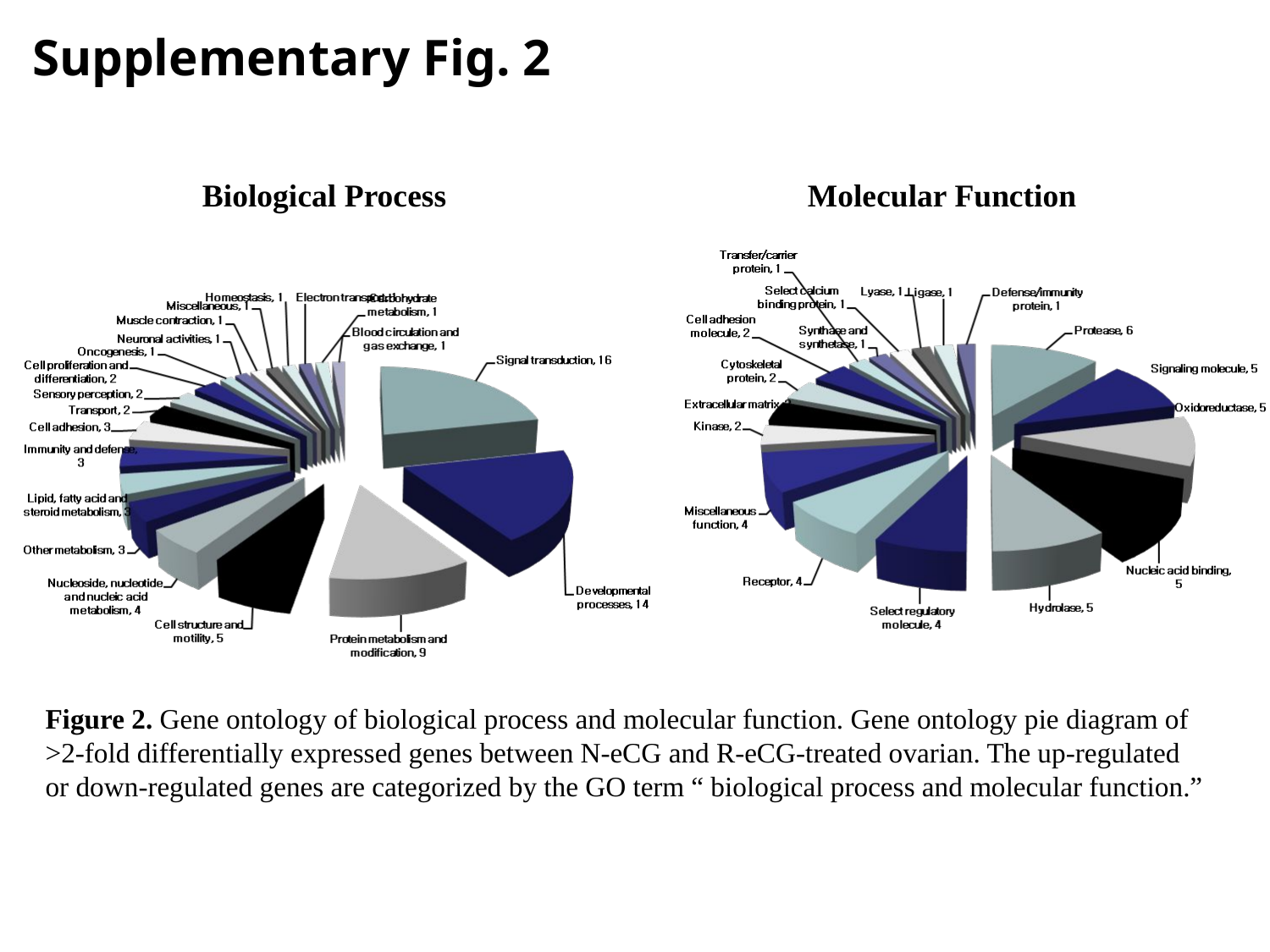

Supplementary Fig. 2
Biological Process
Molecular Function
Figure 2. Gene ontology of biological process and molecular function. Gene ontology pie diagram of
>2-fold differentially expressed genes between N-eCG and R-eCG-treated ovarian. The up-regulated
or down-regulated genes are categorized by the GO term “ biological process and molecular function.”
