# Supplementary material for: Comparative gene expression profiling of mouse ovaries upon stimulation with natural equine chorionic gonadotropin (N-eCG) and tethered recombinant-eCG (R-eCG)
Source: BMC Biotechnol. 2020 Nov 11;20:59. doi: 10.1186/s12896-020-00653-8 (PMC7661263; doi:10.1186/s12896-020-00653-8)
Supplement: Supplementary file 2 — Additional file 2. [file 12896_2020_653_MOESM2_ESM.pptx]

## Slide 1
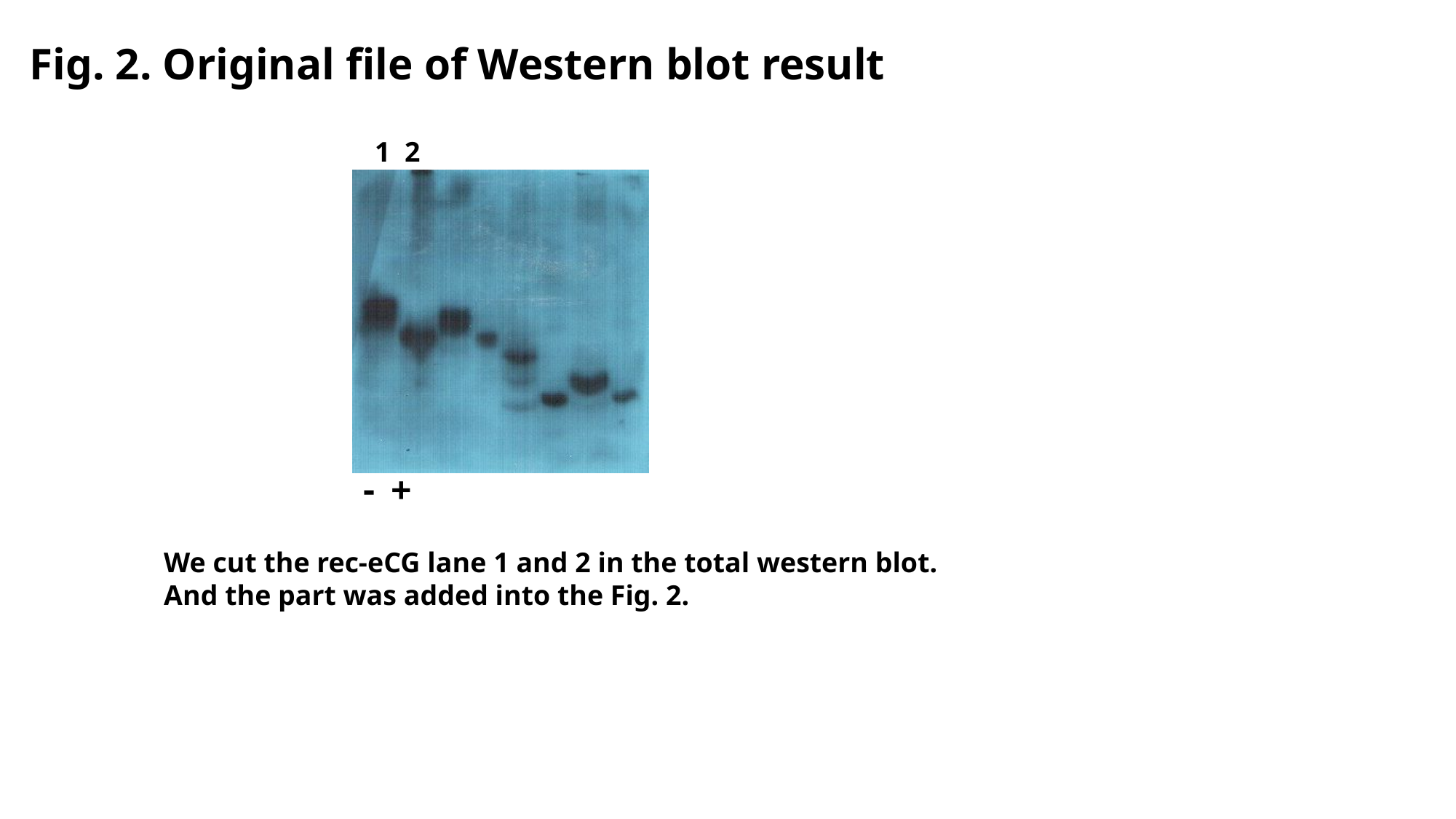

Fig. 2. Original file of Western blot result
1 2
-
+
We cut the rec-eCG lane 1 and 2 in the total western blot.
And the part was added into the Fig. 2.
