# Supplementary material for: Comparative gene expression profiling of mouse ovaries upon stimulation with natural equine chorionic gonadotropin (N-eCG) and tethered recombinant-eCG (R-eCG)
Source: BMC Biotechnol. 2020 Nov 11;20:59. doi: 10.1186/s12896-020-00653-8 (PMC7661263; doi:10.1186/s12896-020-00653-8)
Supplement: Supplementary file 3 — Additional file 3. [file 12896_2020_653_MOESM3_ESM.pptx]

## Slide 1
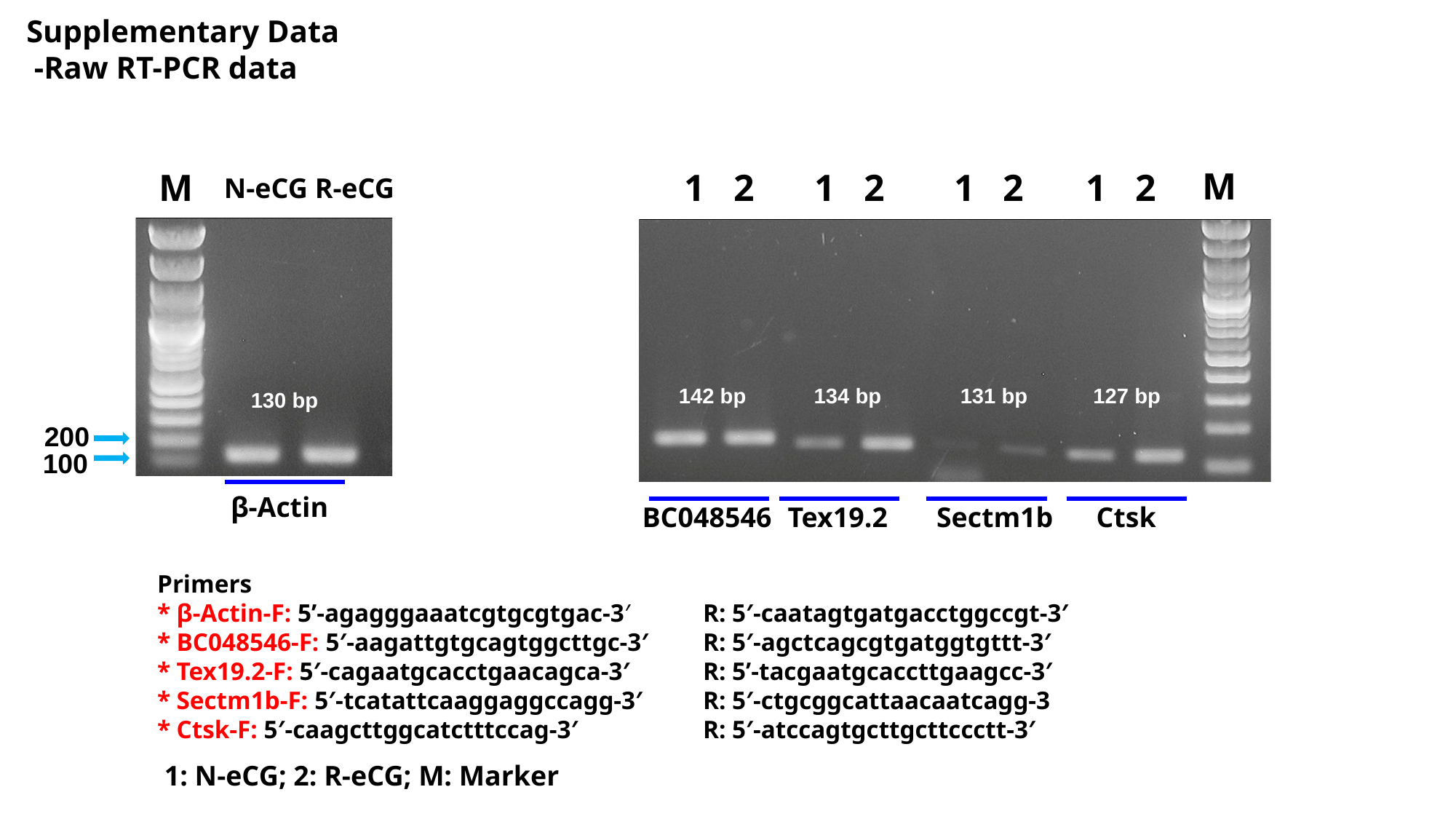

Supplementary Data
 -Raw RT-PCR data
M
M
1 2
1 2
1 2
1 2
N-eCG R-eCG
142 bp
134 bp
131 bp
127 bp
130 bp
200
100
β-Actin
BC048546
Tex19.2
Sectm1b
Ctsk
Primers
* β-Actin-F: 5’-agagggaaatcgtgcgtgac-3′	R: 5′-caatagtgatgacctggccgt-3′
* BC048546-F: 5′-aagattgtgcagtggcttgc-3′ 	R: 5′-agctcagcgtgatggtgttt-3′
* Tex19.2-F: 5′-cagaatgcacctgaacagca-3′	R: 5’-tacgaatgcaccttgaagcc-3′
* Sectm1b-F: 5′-tcatattcaaggaggccagg-3′	R: 5′-ctgcggcattaacaatcagg-3
* Ctsk-F: 5′-caagcttggcatctttccag-3′		R: 5′-atccagtgcttgcttccctt-3′
1: N-eCG; 2: R-eCG; M: Marker

## Slide 2
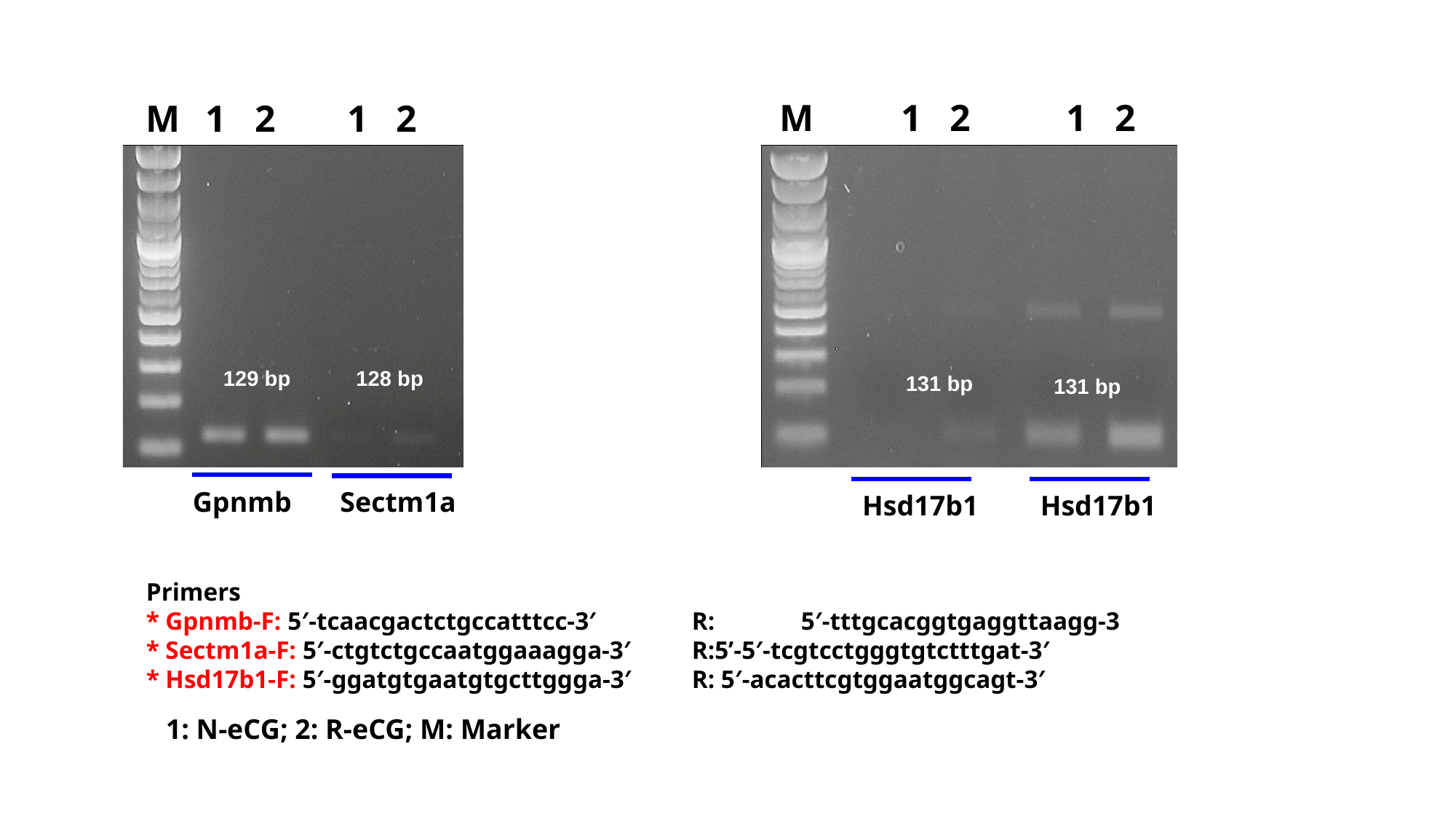

M
1 2
1 2
M
1 2
1 2
129 bp
128 bp
131 bp
131 bp
Gpnmb
Sectm1a
Hsd17b1
Hsd17b1
Primers
* Gpnmb-F: 5′-tcaacgactctgccatttcc-3′	R: 	5′-tttgcacggtgaggttaagg-3
* Sectm1a-F: 5′-ctgtctgccaatggaaagga-3′	R:5’-5′-tcgtcctgggtgtctttgat-3′
* Hsd17b1-F: 5′-ggatgtgaatgtgcttggga-3′	R: 5′-acacttcgtggaatggcagt-3′
1: N-eCG; 2: R-eCG; M: Marker

## Slide 3
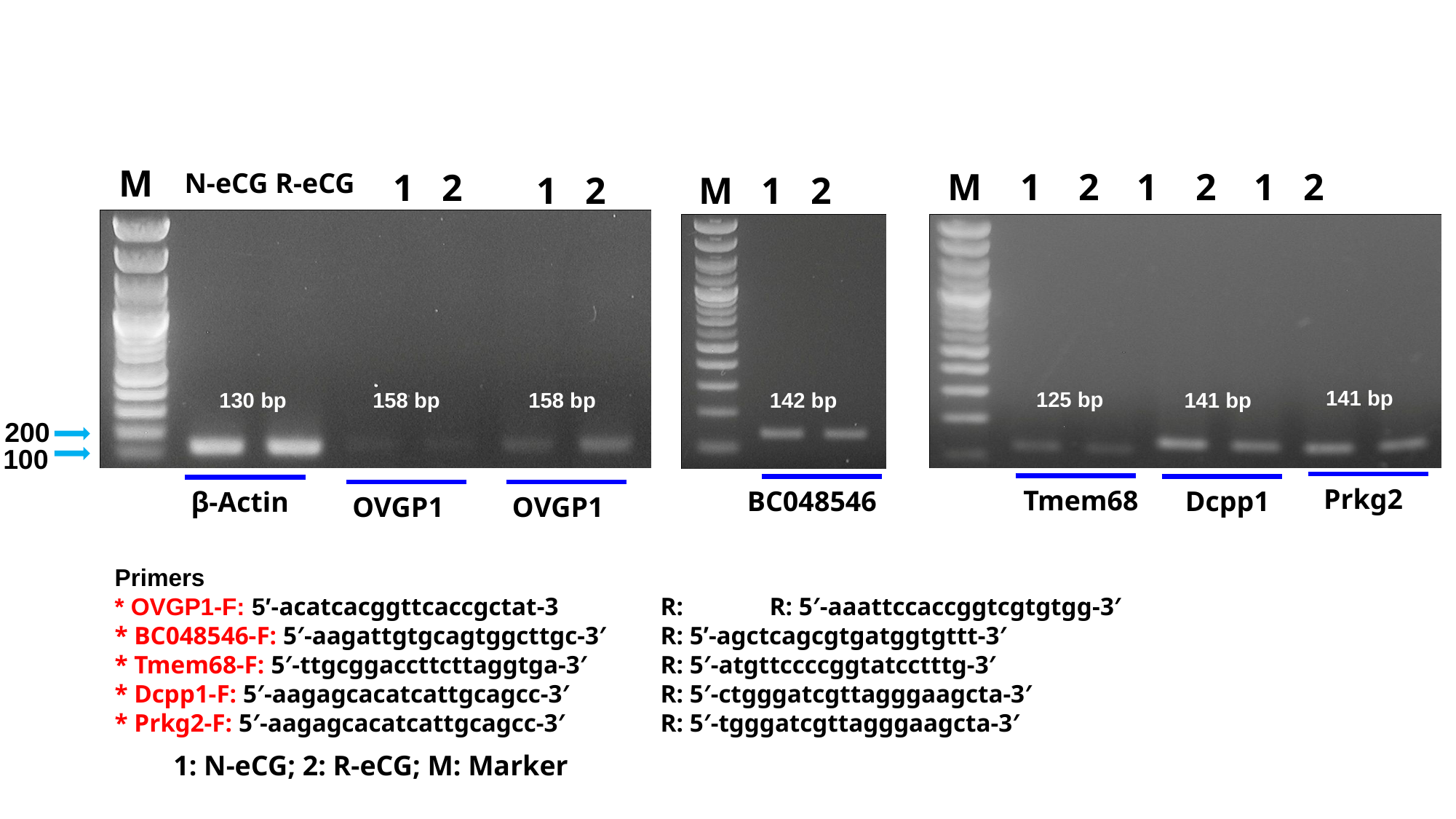

M
M 1 2 1 2 1 2
1 2
N-eCG R-eCG
1 2
M 1 2
141 bp
125 bp
130 bp
158 bp
158 bp
142 bp
141 bp
200
100
Prkg2
Tmem68
BC048546
Dcpp1
β-Actin
OVGP1
OVGP1
Primers
* OVGP1-F: 5′-acatcacggttcaccgctat-3	R: 	R: 5′-aaattccaccggtcgtgtgg-3′
* BC048546-F: 5′-aagattgtgcagtggcttgc-3′	R: 5’-agctcagcgtgatggtgttt-3′
* Tmem68-F: 5′-ttgcggaccttcttaggtga-3′	R: 5′-atgttccccggtatcctttg-3′
* Dcpp1-F: 5′-aagagcacatcattgcagcc-3′	R: 5′-ctgggatcgttagggaagcta-3′
* Prkg2-F: 5′-aagagcacatcattgcagcc-3′	R: 5′-tgggatcgttagggaagcta-3′
1: N-eCG; 2: R-eCG; M: Marker

## Slide 4
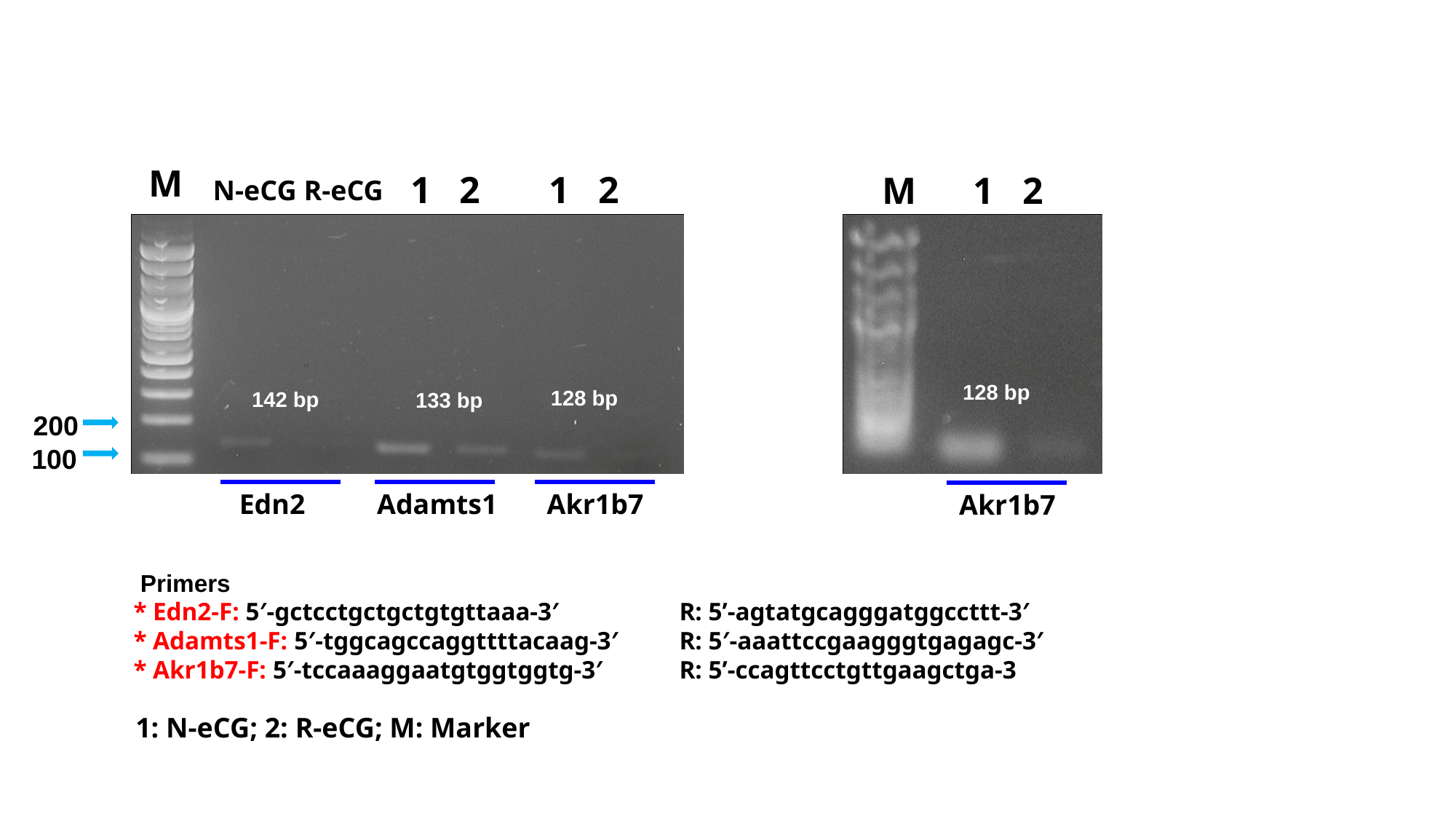

M
1 2
1 2
 M 1 2
N-eCG R-eCG
128 bp
128 bp
142 bp
133 bp
200
100
Edn2
Adamts1
Akr1b7
Akr1b7
 Primers
* Edn2-F: 5′-gctcctgctgctgtgttaaa-3′		R: 5’-agtatgcagggatggccttt-3′
* Adamts1-F: 5′-tggcagccaggttttacaag-3′	R: 5′-aaattccgaagggtgagagc-3′
* Akr1b7-F: 5′-tccaaaggaatgtggtggtg-3′	R: 5’-ccagttcctgttgaagctga-3
1: N-eCG; 2: R-eCG; M: Marker
